# The association between *ATM *D1853N polymorphism and breast cancer susceptibility: a meta-analysis

**DOI:** 10.1186/1756-9966-29-117

**Published:** 2010-08-27

**Authors:** Lin-Bo Gao, Xin-Min Pan, Hong Sun, Xia Wang, Li Rao, Li-Juan Li, Wei-Bo Liang, Mei-Li Lv, Wen-Zhong Yang, Lin Zhang

**Affiliations:** 1 Laboratory of Molecular Translational Medicine, West China Second University Hospital, Sichuan University, Chengdu, Sichuan 610041, China; 2 Department of Forensic Pathology, Henan University of Science and Technology, Luoyang, Henan 471003, China; 3 Department of Forensic Biology, West China School of Preclinical and Forensic Medicine, Sichuan University, Chengdu, Sichuan 610041, China; 4 Department of Immunology, West China School of Preclinical and Forensic Medicine, Sichuan University, Chengdu, Sichuan 610041, China; 5 Department of Cardiology, West China Hospital of Sichuan University, Chengdu, Sichuan, 610041 China; 6 Department of Pathology, College of Preclinical Medicine, Dali University, Dali, Yunnan, 671000, China

## Abstract

**Background:**

Emerging evidence suggests that *ataxia telangiectasia-mutated *(*ATM*) is involved in numerous damage repair signaling pathways and cell-cycle checkpoints. Heterozygous carriers of ATM-mutations have an increased risk for the development of breast cancer. The purpose of this study is to evaluate the association between *ATM *exon39 5557G > A (D1853N, rs1801516) polymorphism and breast cancer susceptibility with the use of a meta-analysis.

**Methods:**

By searching PubMed and Embase databases, a total of 9 epidemiological studies with 4,191 cases and 3,780 controls were identified. Crude odds ratios (ORs) and their corresponding 95% confidence intervals (CIs) for *ATM *D1853N polymorphism and breast cancer risk were calculated using fixed- or random-effects model based on the degree of heterogeneity among studies.

**Results:**

No significant association between the *ATM *D1853N polymorphism and breast cancer risk was observed in overall analysis (GA versus GG: OR = 1.18; 95% CI, 0.90-1.53; AA versus GG: OR = 0.77; 95% CI, 0.58-1.03; dominant model: OR = 1.16; 95% CI, 0.89-1.51; and recessive model: OR = 0.78; 95% CI, 0.59-1.04, respectively).

**Conclusion:**

Our results indicate that *ATM *D1853N polymorphism is not a risk factor for developing breast cancer.

## Background

Ataxia-telangiectasia (A-T) is an autosomal recessive disorder that affects many parts of the body and leads to increased risk of malignancy, including breast cancer [1-3]. A-T is caused by mutations in the *ataxia telangiectasia-mutated *(*ATM*) [4]. ATM, a member of the phosphatidylinositol 3-kinase-like family, plays central roles in the repair of DNA double-strand breaks that was caused by a range of DNA-damaging agents such as ionizing radiation [5].

The *ATM *gene, located on chromosome 11q22-23 and consisting of 66 exons, has been reported to be involved in numerous damage repair signaling pathways and cell-cycle checkpoints [4,6]. Loss of heterozygosity in the region of the *ATM *gene has been detected in approximately 40% of human sporadic breast tumors [7-11]. Breast cancer patients with the combination of radiation treatment and an *ATM *missense variant resulted in a shorter mean interval to develop a second tumor than patients without radiation treatment and *ATM *germline mutation [12]. Previously, some studies reported that female ATM-heterozygous carriers have an increased risk of breast cancer [1,13-18]. In contrast, some studies failed to find that ATM-heterozygous mutations were more frequent in breast cancer cases.

Recently, Mehdipour et al. reported that a common single nucleotide polymorphism *ATM *exon39 5557G > A (D1853N, rs1801516) may be considered as a predisposition factor for developing breast cancer, especially in cancer-prone pedigrees [19]. To date, a number of studies have been performed to investigate the association between the *ATM *D1853N polymorphism and breast cancer risk, but the evidence regarding the role of *ATM *as a genetic marker for breast cancer is conflicting. In order to provide stronger evidence for estimating the association, a meta-analysis was performed.

## Materials and methods

### Eligible studies and data extraction

We searched the articles using the following terms "*ATM*" and "breast cancer" and "polymorphism" or "variant" in PubMed and Embase databases (last search: 31 May, 2010). Additionally, we checked all relevant publications to retrieve the most eligible literatures.

The inclusion criteria were used for the literature selection: (a) articles about *ATM *D1853N polymorphism and breast cancer risk; (b) case-control studies; (c) sufficient published data for calculating odds ratios (ORs) and their corresponding 95% confidence intervals (95% CIs). The following information was collected independently by two investigators (Gao LB and Pan XM) from each study: first author's surname, year of publication, country, ethnicity, number of cases and controls with various genotypes, genotyping techniques, quality control for the genotyping methods, Hardy-Weinberg equilibrium (HWE) and minor allele frequency (MAF) in controls (Table 1).

**Table 1:** Characteristics of literatures included in the meta-analysis

References	Year	Country	Ethnicity	Genotype distribution						HWE (controls)	MAF
						
				case			control				
							
				GG	GA	AA	GG	GA	AA		
Angele [30]	2003	France	European	192	56	6	240	65	7	Yes	0.13
Buchholz [31]	2004	USA	Mixed	39	17	2	394	119	15	Yes	0.14
Dork [32]	2001	Germany	European	753	235	12	422	74	4	Yes	0.08
Gonzalez-Hormazabal [29]	2008	Chile	South American	100	26	0	174	26	0	Yes	0.07
Heikkinen [33]	2005	Finland	European	68	44	9	174	109	23	Yes	0.25
Renwick [34]	2006	UK	European	339	98	6	371	131	19	Yes	0.16
Schrauder [35]	2008	Germany	European	406	99	9	369	129	13	Yes	0.15
Tapia [27]	2008	Chile	South American	74	19	1	183	15	2	No	0.05
Tommiska [36]	2006	Finland	European	954	561	66	404	260	38	Yes	0.24

HWE, Hardy-Weinberg equilibrium

MAF, minor allele frequency

### Statistical analysis

The process of meta-analysis in the current study was performed as described previously in detail [20-22]. In brief, crude ORs and corresponding 95% CIs were preformed to assess the association between *ATM *D1853N polymorphism and breast cancer risk. The pooled ORs were calculated for heterozygote comparison (GA versus GG), homozygote comparison (AA versus GG), dominant model (GA/AA versus GG) and recessive model (AA versus GA/GG), respectively. The statistical heterogeneity among studies was checked by *Q*-test and *I^2 ^*statistics [23]. If the *P *value greater than 0.10 for *Q*-test, indicating absence of heterogeneity, the fixed-effects model (the Mantel-Haenszel method) was used to calculate the pooled OR [24]; otherwise, the random-effects model (the DerSimonian and Laird method) was used [25]. Publication bias of literatures was estimated using Begg's funnel plot [26]. All statistical analyses were carried out with STATA software, version 10.0 (STATA Corp., College Station, TX).

## Results

### Characteristics of studies

Overall, nine studies involving 4,191 cases and 3,780 controls about *ATM *D1853N polymorphism and breast cancer susceptibility were available for this meta-analysis. The main characteristics of eligible studies are summarized in Table 1. There were six studies of European populations, two studies of South American populations, and one study of mixed population that included more than one ethnic descent. Several genotyping methods were used, including polymerase chain reaction-restriction fragment length polymorphism (PCR-RFLP), denatured high performance liquid chromatography (DHPLC), allele-specific oligonucleotide (ASO), PCR-single strand conformation polymorphism (PCR-SSCP), conformation sensitive gel electrophoresis (CSGE), TaqMan, and sequencing. Approximately 67% (6/9) of these studies described quality control for the genotyping assay. The genotype distributions in the controls of all studies were consistent with Hardy-Weinberg equilibrium except for one study [27].

### Main results

The main results of this meta-analysis are shown in Table 2. Overall, no significant association between the *ATM *D1853N polymorphism and breast cancer risk was observed. After subgroup analyses according to ethnicity, significantly increased risk was observed in South American population (GA versus GG: OR = 2.19; 95% CI, 1.38-3.47; and dominant model: OR = 2.15; 95% CI, 1.37-3.38, respectively) but not in European and mixed populations.

**Table 2:** Main results of pooled ORs in the meta-analysis

	n^a^	Cases/controls	GA versus GG		AA versus GG		GA/AA versus GG (dominant)		AA versus GA/GG (recessive)	
						
			OR (95%CI)	*P *^b^	OR (95%CI)	*P *^b^	OR (95%CI)	*P *^b^	OR (95%CI)	*P *^b^
Total	9	4,191/3,780	1.18 (0.90-1.53)	< 0.001	0.77 (0.58-1.03)	0.50	1.16 (0.89-1.51)	< 0.001	0.78 (0.59-1.04)	0.66
Ethnicity										
European	6	3,913/2,852	1.00 (0.77-1.31)	< 0.001	0.75 (0.56-1.01)	0.34	0.98 (0.75-1.29)	< 0.001	0.77 (0.57-1.02)	0.46
South American	2	220/400	2.19 (1.38-3.47)	0.22	1.24 (0.11-13.84)	-	2.15 (1.37-3.38)	0.27	1.07 (0.10-11.89)	-
Mixed	1	58/528	1.44 (0.79-2.64)	-	1.35 (0.30-6.11)	-	1.43 (0.80-2.56)	-	1.22 (0.27-5.48)	-

^a ^Number of comparisons

^b ^*P *value of Q-test for heterogeneity test. Random-effects model was used if the *P *value <0.10; otherwise, fixed-effects model was used

### Publication bias

Begg's funnel plot was used to identify the potential publication bias of literatures on breast cancer, and the results did not show any evidence of publication bias in any comparison model (*P *> 0.05).

## Discussion

Previous studies have inconclusive results about the association between *ATM *D1853N polymorphism and breast cancer risk, which might be caused by relatively small sample size in a single study. Meta-analysis offers a rational and helpful way to solve this practical problem by combination the findings from independent studies. In the current meta-analysis, we cumulated the data from nine case-control studies to explore the association between *ATM *D1853N polymorphism and breast cancer risk. No significant association between this polymorphism and breast cancer risk was observed in the overall study populations. Our result was consistent with the finding from a previous meta-analysis showing that another polymorphism of *ATM *(S49C, rs1800054) was not significantly associated with breast cancer susceptibility [28]. This finding indicates that the *ATM *D1853N polymorphism is not a risk factor for developing breast cancer, although a significantly increased risk of breast cancer in ATM-heterozygous carriers has been reported [1,13-18].

After subgroup analyses according to ethnicity, we found that the *ATM *D1853N polymorphism was associated with a significantly increased risk of breast cancer in South American population (heterozygote comparison and dominant model) but not in European and mixed populations. The reason for these discrepancies is not very clear. There are, however, some possible reasons. Firstly, the *ATM *D1853N polymorphism may present with different frequencies in different populations and as a result may be associated with different degrees of breast cancer risk among different ethnic populations. Secondly, the genotype distribution in the controls of a South American study was departed from Hardy-Weinberg equilibrium [27], indicating that there was a high risk of selection bias because the controls may not be representative of the general population very well. Thirdly, the positive association might have occurred by chance due to the insufficient statistical power with only two South American studies eligible in this meta-analysis [27,29]. Therefore, additional studies with larger sample size are of great importance to clarify this finding.

Some limitations of this meta-analysis should be taken into consideration. On the one hand, the numbers of cases and controls analyzed for D1853N (rs1801516) found in the literature is still very small and might not precisely answer the given question. Especially, for some subgroup analyses, the statistical power is so low that caution should be taken in interpreting these results, even though positive association was found in South American population. On the other hand, data were not stratified by age at menarche, number of full-term pregnancies, menopausal status, and other suspected factors due to absence of available information.

In conclusion, the overall outcomes of this meta-analysis have shown that the *ATM *D1853N polymorphism is not associated with breast cancer risk, indicating that this polymorphism is not an independent risk factor for the development of breast cancer. Well-designed, unbiased studies with a wider spectrum of subjects should be of great value to explore other potential risk factors.

## Competing interests

The authors declare that they have no competing interests.

## Authors' contributions

GLB, PXM, and Zhang L designed the study, and wrote the manuscript; SH, WX, and RL performed data acquisition; LLJ performed quality control of data; LWB, LML, and YWZ performed statistical analysis and interpretation. All authors read and approved the final manuscript.

## References

[B1] Swift M Reitnauer PJ Morrell D Chase CL Breast and other cancers in families with ataxia-telangiectasia N Engl J Med 1987 316 1289 1294 10.1056/NEJM198705213162101 3574400

[B2] Chen J Birkholtz GG Lindblom P Rubio C Lindblom A The role of ataxia-telangiectasia heterozygotes in familial breast cancer Cancer Res 1998 58 1376 1379 9537233

[B3] Borresen AL Andersen TI Tretli S Heiberg A Moller P Breast cancer and other cancers in Norwegian families with ataxia-telangiectasia Genes Chromosomes Cancer 1990 2 339 340 10.1002/gcc.2870020412 2268581

[B4] Savitsky K Bar-Shira A Gilad S Rotman G Ziv Y Vanagaite L Tagle DA Smith S Uziel T Sfez S Ashkenazi M Pecker I Frydman M Harnik R Patanjali SR Simmons A Clines GA Sartiel A Gatti RA Chessa L Sanal O Lavin MF Jaspers NG Taylor AM Arlett CF Miki T Weissman SM Lovett M Collins FS Shiloh Y A single ataxia telangiectasia gene with a product similar to PI-3 kinase Science 1995 268 1749 1753 10.1126/science.7792600 7792600

[B5] Abraham RT PI 3-kinase related kinases: 'big' players in stress-induced signaling pathways DNA Repair (Amst) 2004 3 883 887 10.1016/j.dnarep.2004.04.002 15279773

[B6] Shiloh Y Kastan MB ATM: genome stability, neuronal development, and cancer cross paths Adv Cancer Res 2001 83 209 254 full_text 11665719 10.1016/s0065-230x(01)83007-4

[B7] Angele S Hall J The ATM gene and breast cancer: is it really a risk factor? Mutat Res 2000 462 167 178 10.1016/S1383-5742(00)00034-X 10767628

[B8] Negrini M Rasio D Hampton GM Sabbioni S Rattan S Carter SL Rosenberg AL Schwartz GF Shiloh Y Cavenee WK Croce CM Definition and refinement of chromosome 11 regions of loss of heterozygosity in breast cancer: identification of a new region at 11q23.3 Cancer Res 1995 55 3003 3007 7606718

[B9] Laake K Launonen V Niederacher D Gudlaugsdottir S Seitz S Rio P Champeme MH Bieche I Birnbaum D White G Sztan M Sever N Plummer S Osorio A Broeks A Huusko P Spurr N Borg A Cleton-Jansen AM van't Veer L Benitez J Casey G Peterlin B Olah E Borresen-Dale AL Loss of heterozygosity at 11q23.1 and survival in breast cancer: results of a large European study. Breast Cancer Somatic Genetics Consortium Genes Chromosomes Cancer 1999 25 212 221 10.1002/(SICI)1098-2264(199907)25:3<212::AID-GCC3>3.0.CO;2-G 10379867

[B10] Hampton GM Mannermaa A Winqvist R Alavaikko M Blanco G Taskinen PJ Kiviniemi H Newsham I Cavenee WK Evans GA Loss of heterozygosity in sporadic human breast carcinoma: a common region between 11q22 and 11q23.3 Cancer Res 1994 54 4586 4589 8062246

[B11] Carter SL Negrini M Baffa R Gillum DR Rosenberg AL Schwartz GF Croce CM Loss of heterozygosity at 11q22-q23 in breast cancer Cancer Res 1994 54 6270 6274 7954477

[B12] Broeks A Braaf LM Huseinovic A Schmidt MK Russell NS van Leeuwen FE Hogervorst FB van't Veer LJ The spectrum of ATM missense variants and their contribution to contralateral breast cancer Breast Cancer Res Treat 2008 107 243 248 10.1007/s10549-007-9543-6 17393301 PMC2137941

[B13] Swift M Morrell D Massey RB Chase CL Incidence of cancer in 161 families affected by ataxia-telangiectasia N Engl J Med 1991 325 1831 1836 10.1056/NEJM199112263252602 1961222

[B14] Easton DF Cancer risks in A-T heterozygotes Int J Radiat Biol 1994 66 S177 182 10.1080/09553009414552011 7836845

[B15] Inskip HM Kinlen LJ Taylor AM Woods CG Arlett CF Risk of breast cancer and other cancers in heterozygotes for ataxia-telangiectasia Br J Cancer 1999 79 1304 1307 10.1038/sj.bjc.6690209 10098776 PMC2362264

[B16] Athma P Rappaport R Swift M Molecular genotyping shows that ataxia-telangiectasia heterozygotes are predisposed to breast cancer Cancer Genet Cytogenet 1996 92 130 134 10.1016/S0165-4608(96)00328-7 8976369

[B17] Broeks A Urbanus JH Floore AN Dahler EC Klijn JG Rutgers EJ Devilee P Russell NS van Leeuwen FE van't Veer LJ ATM-heterozygous germline mutations contribute to breast cancer-susceptibility Am J Hum Genet 2000 66 494 500 10.1086/302746 10677309 PMC1288102

[B18] Milne RL Variants in the ATM gene and breast cancer susceptibility Genome Medicine 2009 1 10.1186/gm12 19348699 PMC2651585

[B19] Mehdipour P Mahdavi M Mohammadi-Asl J Atri M Importance of ATM gene as a susceptible trait: predisposition role of D1853N polymorphism in breast cancer Medical Oncology 2010 1 5 20396981 10.1007/s12032-010-9525-0

[B20] Gao LB Pan XM Jia J Liang WB Rao L Xue H Zhu Y Li SL Lv ML Deng W Chen TY Wei YG Zhang L IL-8 -251A/T polymorphism is associated with decreased cancer risk among population-based studies: evidence from a meta-analysis Eur J Cancer 2010 46 1333 1343 10.1016/j.ejca.2010.03.011 20400292

[B21] Gao LB Bai P Pan XM Jia J Li LJ Liang WB Tang M Zhang LS Wei YG Zhang L The association between two polymorphisms in pre-miRNAs and breast cancer risk: a meta-analysis Breast Cancer Res Treat 2010 in press 10.1007/s10549-010-0993-x 20640596

[B22] Gao LB Pan XM Li LJ Liang WB Zhu Y Zhang LS Wei YG Tang M Zhang L RAD51 135G/C polymorphism and breast cancer risk: a meta-analysis from 21 studies Breast Cancer Res Treat 2010 in press 10.1007/s10549-010-0995-8 20640595

[B23] Higgins JP Thompson SG Quantifying heterogeneity in a meta-analysis Stat Med 2002 21 1539 1558 10.1002/sim.1186 12111919

[B24] Mantel N Haenszel W Statistical aspects of the analysis of data from retrospective studies of disease J Natl Cancer Inst 1959 22 719 748 13655060

[B25] DerSimonian R Laird N Meta-analysis in clinical trials Control Clin Trials 1986 7 177 188 10.1016/0197-2456(86)90046-2 3802833

[B26] Egger M Davey Smith G Schneider M Minder C Bias in meta-analysis detected by a simple, graphical test BMJ 1997 315 629 634 9310563 10.1136/bmj.315.7109.629 PMC2127453

[B27] Tapia T Sanchez A Vallejos M Alvarez C Moraga M Smalley S Camus M Alvarez M Carvallo P ATM allelic variants associated to hereditary breast cancer in 94 Chilean women: susceptibility or ethnic influences? Breast Cancer Res Treat 2008 107 281 288 10.1007/s10549-007-9544-5 17351744

[B28] Cox A Dunning AM Garcia-Closas M Balasubramanian S Reed MW Pooley KA Scollen S Baynes C Ponder BA Chanock S Lissowska J Brinton L Peplonska B Southey MC Hopper JL McCredie MR Giles GG Fletcher O Johnson N dos Santos Silva I Gibson L Bojesen SE Nordestgaard BG Axelsson CK Torres D Hamann U Justenhoven C Brauch H Chang-Claude J Kropp S Risch A Wang-Gohrke S Schurmann P Bogdanova N Dork T Fagerholm R Aaltonen K Blomqvist C Nevanlinna H Seal S Renwick A Stratton MR Rahman N Sangrajrang S Hughes D Odefrey F Brennan P Spurdle AB Chenevix-Trench G Beesley J Mannermaa A Hartikainen J Kataja V Kosma VM Couch FJ Olson JE Goode EL Broeks A Schmidt MK Hogervorst FB Van't Veer LJ Kang D Yoo KY Noh DY Ahn SH Wedren S Hall P Low YL Liu J Milne RL Ribas G Gonzalez-Neira A Benitez J Sigurdson AJ Stredrick DL Alexander BH Struewing JP Pharoah PD Easton DF A common coding variant in CASP8 is associated with breast cancer risk Nat Genet 2007 39 352 358 10.1038/ng1981 17293864

[B29] Gonzalez-Hormazabal P Bravo T Blanco R Valenzuela CY Gomez F Waugh E Peralta O Ortuzar W Reyes JM Jara L Association of common ATM variants with familial breast cancer in a South American population BMC Cancer 2008 8 117 10.1186/1471-2407-8-117 18433505 PMC2386480

[B30] Angele S Romestaing P Moullan N Vuillaume M Chapot B Friesen M Jongmans W Cox DG Pisani P Gerard JP Hall J ATM haplotypes and cellular response to DNA damage: association with breast cancer risk and clinical radiosensitivity Cancer Res 2003 63 8717 8725 14695186

[B31] Buchholz TA Weil MM Ashorn CL Strom EA Sigurdson A Bondy M Chakraborty R Cox JD McNeese MD Story MD A Ser49Cys Variant in the Ataxia Telangiectasia, Mutated, Gene that Is More Common in Patients with Breast Carcinoma Compared with Population Controls Cancer 2004 100 1345 1351 10.1002/cncr.20133 15042666

[B32] Dork T Bendix R Bremer M Rades D Klopper K Nicke M Skawran B Hector A Yamini P Steinmann D Weise S Stuhrmann M Karstens JH Spectrum of ATM gene mutations in a hospital-based series of unselected breast cancer patients Cancer Res 2001 61 7608 7615 11606401

[B33] Heikkinen K Rapakko K Karppinen SM Erkko H Nieminen P Winqvist R Association of common ATM polymorphism with bilateral breast cancer Int J Cancer 2005 116 69 72 10.1002/ijc.20996 15756685

[B34] Renwick A Thompson D Seal S Kelly P Chagtai T Ahmed M North B Jayatilake H Barfoot R Spanova K McGuffog L Evans DG Eccles D Easton DF Stratton MR Rahman N ATM mutations that cause ataxia-telangiectasia are breast cancer susceptibility alleles Nat Genet 2006 38 873 875 10.1038/ng1837 16832357

[B35] Schrauder M Frank S Strissel PL Lux MP Bani MR Rauh C Sieber CC Heusinger K Hartmann A Schulz-Wendtland R Strick R Beckmann MW Fasching PA Single nucleotide polymorphism D1853N of the ATM gene may alter the risk for breast cancer J Cancer Res Clin Oncol 2008 134 873 882 10.1007/s00432-008-0355-9 18264724 PMC12160744

[B36] Tommiska J Jansen L Kilpivaara O Edvardsen H Kristensen V Tamminen A Aittomaki K Blomqvist C Borresen-Dale AL Nevanlinna H ATM variants and cancer risk in breast cancer patients from Southern Finland BMC Cancer 2006 6 209 10.1186/1471-2407-6-209 16914028 PMC1592307

